# Novel Algicides against Bloom-Forming Cyanobacteria from Allelochemicals: Design, Synthesis, Bioassay, and 3D-QSAR Study

**DOI:** 10.3390/biology10111145

**Published:** 2021-11-06

**Authors:** Yin Luo, Yushun Yang, Wenguang Hou, Jie Fu

**Affiliations:** 1 Department of Biomedical Engineering, School of Life Science and Technology, Huazhong University of Science and Technology, Wuhan 430074, China; 2019511006@hust.edu.cn; 2 School of Environmental Science and Engineering, Huazhong University of Science and Technology, Wuhan 430074, China; 3 State Key Laboratory of Pharmaceutical Biotechnology, School of Life Sciences, Nanjing University, Nanjing 210093, China; ys_yang@nju.edu.cn

**Keywords:** cyanobacteria, allelochemicals, algicide, phenolic acid, 3D-QSAR

## Abstract

**Simple Summary:**

Due to the frequent outbreaks of cyanobacteria bloom worldwide, research on novel algicides has attracted more and more attention. At present, allelochemicals have been reported as promising natural algicides. However, current studies mainly focus on the parent compounds, and the structural modification of original allelochemicals has been rarely involved. In this study, phenolic acid derivatives were innovatively synthesized as potential algicides, and lead compounds with excellent activity were found. For instance, upon the algicidal activity on *Aphanizomenon flos-aquae*, the EC_50_ of the best active compound **18** reached 0.63 µM (0.17 mg/L), while the EC_50_ values of previously reported allelochemicals have been basically at the mg/L level. The result indicates that the algicides reported in this study are more efficient at inhibiting cyanobacteria with lower effective concentrations than most previously reported compounds. Moreover, 3D-QSAR models were constructed and provided a theoretical guidance for further structure optimization of compounds to achieve better algicidal activity.

**Abstract:**

Cyanobacteria bloom caused by water eutrophication has threatened human health and become a global environmental problem. To develop green algicides with strong specificity and high efficiency, three series of ester and amide derivatives from parent allelochemicals of caffeic acid (CA), cinnamic acid (CIA), and 3-hydroxyl-2-naphthoic acid (HNA) were designed and synthesized. Their inhibitory effects on the growth of five harmful cyanobacterial species, *Microcystis aeruginosa* (*M. aeruginosa*), *Microcystis wesenbergii* (*M. wesenbergii*), *Microcystis flos-aquae* (*M. flos-aquae*), *Aphanizomenon flos-aquae* (*Ap. flos-aquae*), and *Anabaena flos-aquae* (*An. flos-aquae*), were evaluated. The results revealed that CIA esters synthesized by cinnamic acid and fatty alcohols showed the best inhibition effect, with EC_50_ values ranging from 0.63 to >100 µM. Moreover, some CIA esters exhibited a good selectivity in inhibiting cyanobacteria. For example, the inhibitory activity of naphthalen-2-yl cinnamate was much stronger on *Ap. flos-aquae* (EC_50_ = 0.63 µM) than other species (EC_50_ > 10 µM). Three-dimensional quantitative structure–activity relationship (3D-QSAR) analysis was performed and the results showed that the steric hindrance of the compounds influenced the algicidal activity. Further mechanism study found that the inhibition of CIA esters on the growth of *M. aeruginosa* might be related to the accumulation of malondialdehyde (MDA).

## 1. Introduction

With the increase of human activities, water eutrophication has become a prominent and global problem [1]. Eutrophic water is rich in nitrogen, phosphorus, and other nutrients, which leads to the excessive propagation of harmful blue-green algae (cyanobacteria) and the formation of cyanobacteria bloom [2,3]. With the impact of cyanobacteria bloom, the dissolved oxygen content in the water decreases, a large number of fish and other organisms die, and the whole ecosystem is seriously damaged. Cyanobacteria can also produce a variety of toxins (such as microcystin, nodularin, and cylindropermopsin), which may threaten human health [4]. How to inhibit the growth of cyanobacteria has become a hot topic in the field of environmental protection and the common measure is to apply chemical algicides. However, the application of algicides has some limitations, including easily causing secondary pollution and lack of enough inhibition specificity on target algae [5]. Therefore, the development of green algicides with strong specificity and high efficiency is urgently needed.

Allelochemicals refer to the chemical substances released from plants or microorganisms that affect the growth of adjacent plants or microorganisms [6,7]. Previous studies showed that organic acids and their derivatives extracted from plants have strong inhibitory effects on algal growth [8]. Caffeic acid (CA), cinnamic acid (CIA), and naphthoic acid (NA), as common allelochemicals extracted from plants, have been reported to inhibit the growth of some algae [8,9,10]. He et al. [11] found that CIA had an inhibitory effect on *Microcystis aeruginosa* (*M. aeruginosa*), a dominant species in cyanobacteria bloom, with an EC_50_ of 0.487 mmol/L. Gao et al. [12] extracted four phenolic acids, including CA, from *Hydrilla verticillata* and *Vallisneria spiralis*, and found that the four substances had synergetic inhibition on *M. aeruginosa*. These positive results encourage us to find more efficient green algicides from the derivatives of organic acids.

In addition, the species diversity of cyanobacteria increases the difficulty of controlling cyanobacteria bloom by using algicides. For example, there are 12 species from *Microcystis* and 13 species from *Anabaena* contributing to the cyanobacteria bloom in Taihu Lake, China [13]. The dominant species of cyanobacteria in Taihu Lake are *Microcystis* spp., while the dominant species in Wuli Lake, an inner lake of Taihu Lake, are filamentous cyanobacteria. When cyanobacteria bloom breaks out, dominant species may change with the seasonal succession [14]. The succession rule of the cyanobacteria in Taihu Lake was found as: *Microcystis flos-aquae* (*M. flos-aquae*) was dominant in spring, *Microcystis wesenbergii* (*M. wesenbergii*) in summer, *M. wesenbergii* and *M. aeruginosa* in autumn, and *M. flos-aquae* and *Microcystis panniformis* in winter [13]. Therefore, it is necessary to apply efficient and specific algicides for the control of cyanobacteria bloom with consideration of the dominant species as well as the variation in different seasons.

In this study, three series of ester and amide derivatives were designed and synthesized from the parent compounds: CIA, CA, and 3-hydroxy-2-naphthoic acid (HNA). Their inhibitory effects on the growth of five harmful cyanobacteria species with toxin production capacity [15,16,17,18], *M. aeruginosa*, *M. wesenbergii*, *M. flos-aquae*, *Aphanizomenon flos-aquae* (*Ap. flos-aquae*), and *Anabaena flos-aquae* (*An. flos-aquae*), were evaluated. The structure–activity relationship was discussed, and three-dimensional quantitative structure–activity relationship (3D-QSAR) analysis was performed. Moreover, physiological assays for examination of the total soluble protein content, superoxide dismutase (SOD) activity, and malondialdehyde (MDA) content were performed to explore possible action mechanisms. The purpose of this study was to find efficient and specific lead compounds of green algicides for the control of cyanobacteria bloom.

## 2. Materials and Methods

### 2.1. Chemistry General

All used chemicals of reagent grade were purchased from Aldrich (USA). Separation of the compounds by column chromatography was carried out with silica gel 60 (200–300 mesh ASTM, E. Merck, Darmstadt, Germany). The quantity of silica gel used was 50–100 times the weight charged on the column. Thin-layer chromatography (TLC) was run on the silica gel-coated aluminum sheets (silica gel 60 GF254, E. Merck, Darmstadt, Germany) and visualized under UV light (254 nm). Melting points (uncorrected) were obtained by using an XT4 MP apparatus (Taike Corp. Beijing, China). ^1^H NMR spectra were recorded at 300 MHz on ^1^H-Varian-Mercury-300 spectrometers at 25 °C, using tetramethylsilane (TMS) as internal standard. ESI MS were recorded with a Mariner System 5304 mass spectrometer. Elementary analyses were performed on a CHN-O-Rapid instrument.

### 2.2. Synthesis of Caffeic Acid Esters

**Scheme 1:**
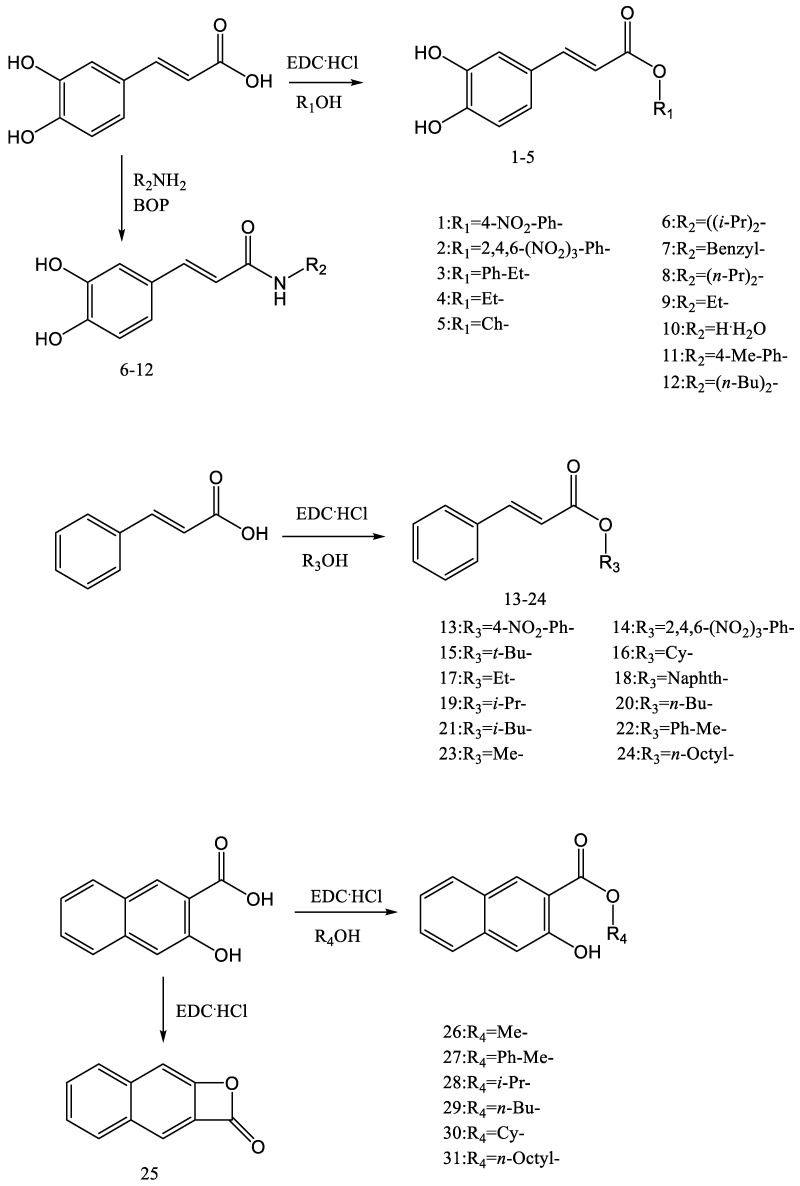
Synthesis route of compounds **1**–**31**.

Caffeic acid (2.5 mmol) was dissolved in 5 mL of *N*,*N*-dimethylformamide (DMF). Alcohol or phenol (2.5 mmol) was added, following by 10 mL of CH_2_Cl_2_ solution of 1-ethyl-3-(3-dimethylaminopropyl) carbodiimide hydrochloride (EDC∙HCl, 3 mmol). The mixture was stirred at room temperature for 6–10 h. CH_2_Cl_2_ was removed under reduced pressure and the solution was diluted using 40 mL of water. The products were extracted using ethyl acetate (EtOAc). The extract was dried using MgSO_4_, filtered, and evaporated. The residue was purified on a silica gel column (EtOAc-petroleum ether (peth)) to give CA esters (**1**–**5**). The synthesis route of compounds **1**–**5** is shown in Scheme 1. The characterization data for compounds **1**–**5** are given in the Supplementary Materials S1.

### 2.3. Synthesis of Caffeic Acid Amides

Caffeic acid (5 mmol) was dissolved in 10 mL of DMF, and 0.7 mL of triethanolamine (TEA) (5 mmol) were added. The solution was cooled in an ice-water bath, and amine (5 mmol) was added, following by 10 mL of CH_2_Cl_2_ solution of butyloctyl phthalate (BOP, 5 mmol). The mixture was stirred at 0 °C for 30 min and then at room temperature for 2–5 h. CH_2_Cl_2_ was removed under reduced pressure and the solution was diluted with 80 mL of water. The products were extracted with EtOAc. The extract was washed successively with 1 N HCl, water, 1 M NaHCO_3_, and water, dried using MgSO_4_, filtered and evaporated. The residue was purified on a silica gel column (EtOAc-peth) to give CA amides (**6**–**12**). The synthesis route of compounds **6**–**12** is shown in Scheme 1. The characterization data for compounds **6**–**12** are given in the Supplementary Materials S1.

### 2.4. Synthesis of Cinnamic Acid Esters

Cinnamic acid (2.5 mmol) was dissolved in 10 mL of CH_2_Cl_2_. Alcohol or phenol (2.5 mmol) was added, following by 10 mL of CH_2_Cl_2_ solution of EDC∙HCl (3 mmol). The mixture was stirred at room temperature for 6–10 h. Then, the solution was dried using MgSO_4_, filtered, and evaporated. The residue was purified on a silica gel column (EtOAc-peth) to give CIA esters (**13**–**24**). The synthesis route of compounds **13**–**24** is shown in Scheme 1. The characterization data for compounds **13**–**24** are given in the Supplementary Materials S1.

### 2.5. Synthesis of 2-Hydroxy-3-Naphthoic Acid Esters

The method was the same as that of the synthesis of CIA esters (**26**–**31**). **25** was the main by-product during the reaction via lactonization of 2-hydroxy-3-naphthoic acid. The synthesis route of compounds **26**–**31** is shown in Scheme 1. The characterization data for compounds **25**–**31** are given in the Supplementary Materials S1.

### 2.6. Algicidal Activity Assay

*M. aeruginosa*, *M. wesenbergii*, *M. flos-aquae*, *Ap. flos-aquae*, and *An. flos-aquae* were provided by the Freshwater Algae Culture Collection of the Institute of Hydrobiology, Chinese Academy of Sciences (Wuhan, China). The culture conditions were as follows: BG11 medium (25 mL, Erlenmeyer flasks), 12-h light/12-h dark cycle, and 25 °C. The flasks were shaken 3 times/day, making cyanobacterial cells grow into the logarithmic phase. For the algicidal activity assay, different doses (0, 1, 10, and 100 μmol/L) of compounds were added into 25 mL of sterilized BG11 culture medium, and the solution was mixed uniformly by hand. Then, the cyanobacterium was inoculated into the culture medium with an initial density of 10^6^ cell/L, and cultured for 96 h with shaking twice in the daytime. Then, 0.5 mL of uniform cyanobacteria solution were withdrawn and the algal density was counted. EC_50_ was calculated based on the dose–response experiments by the probit unit method using the software SPSS.

### 2.7. Physiological Assays

At the end of the algicidal experiments on compound 24 and CIA, the *M. aeruginosa* cells were harvested, and the cell pellets were collected after centrifugation, and subjected to the measurements of the following assays. The content of total soluble protein was determined by the Coomassie brilliant blue G-250 staining method [19]. An appropriate amount of algal cell pellets was taken, resuspended by phosphate buffer saline (PBS), and broken by ultrasonication in an ice bath. The supernatant was taken after centrifugation at 4 °C. Coomassie brilliant blue G250 dye solution was added into the supernatant, and the supernatant was shaken and mixed completely. A spectrophotometer was used to detect the absorbance of the prepared solution at 595 nm.

The activity of superoxide dismutase (SOD) was determined by the WST-1 method [20], using the SOD assay kit provided by Nanjing Jiancheng Bioengineering Institute (Nanjing, China) and strictly according to the manufacturer’s instructions.

The content of malondialdehyde (MDA) in cells was determined by thiobarbituric acid colorimetry [21]. An appropriate amount of algal cell pellets was taken, resuspended by PBS, and broken by ultrasonication in an ice bath. The supernatant was taken after centrifugation and stored at −20 °C for subsequent operation. MDA was measured by the MDA assay kit obtained from Abcam (Cambridge, MA, USA).

### 2.8. 3D-QSAR Model Study

All 34 compounds including synthesized compounds (**1**–**31**) and parent compounds (CIA, CA, and HNA) were utilized as a training set for 3D-QSAR modeling. Compounds **1**, **3**, **10**, **16**, **20**, **25**, and **31** were randomly selected as an external test set for validation of the constructed models. The inhibitory ability of the compounds [EC_50_ (µmol/L)] was changed to a negative logarithmic scale [pEC_50_ (µmol/L)], and then used for subsequent QSAR analyses as a response variable. The operation was performed based on the comparative molecular field analysis (CoMFA) method by Discovery Studio 3.5. The whole modeling process was referenced to the previous publication [22].

### 2.9. Statistical Analysis

All the experiments were conducted in triplicate. Dunnett’s multiple comparisons test, following one-way analysis of variance, was used to statistically analyze the data using GraphPad Prism version 7.0 software for Windows (USA). Values were considered significant when *p* < 0.05. Principal co-ordinates analysis (PCoA) was performed using vegan package in R version 4.0 to explore the characteristics of compounds and cyanobacteria based on the data of the algicidal activity.

## 3. Results

### 3.1. Algicidal Activity of Synthesized Compounds

**Figure 1:**
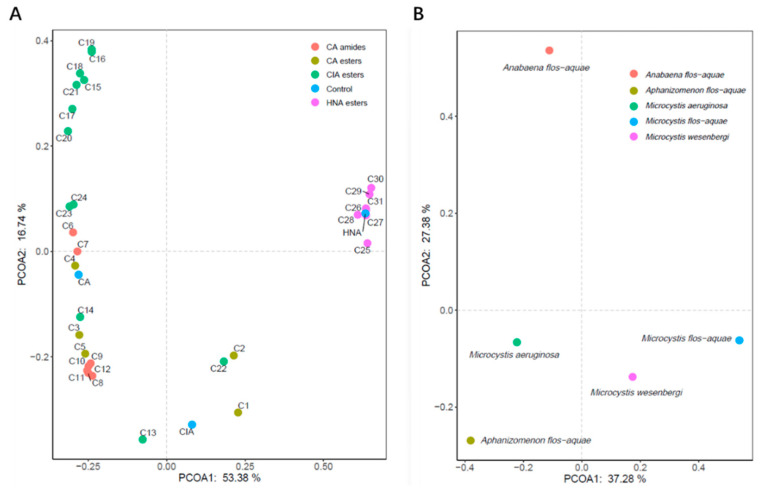
Scatter plots of the first and second principal coordinates of PCoA. (**A**): PCoA analysis based on compounds. (**B**): PCoA analysis based on cyanobacteria.

The algicidal activity of the synthesized compounds **1**–**31** against *M. aeruginosa*, *M. wesenbergii*, *M. flos-aquae*, *Ap. flos-aquae*, and *An. flos-aquae* was evaluated with the parent compounds CA, CIA, and HNA as positive controls. The EC_50_ data of each compound are summarized in Table 1. To help explain the results, we carried out PCoA based on the compounds and cyanobacteria, respectively, and the two-dimensional scatter plots are shown in Figure 1.

Generally, of all synthesized compounds, CIA esters exhibited better algicidal effects than other compound series. Compounds **15**–**21** from CIA esters, synthesized by cinnamic acid and fatty alcohols, showed strong activities against these cyanobacteria except *An. flos-aquae* with EC_50_ values of 0.63–24.51 µmol/L. The scatter plot of PCoA also showed these compounds were gathered in the upper left corner (Figure 1A); the second axis seemed to sort the compounds by algicidal activity, and the large loading value presented a higher algicidal activity. The activities of most CIA esters against *M. wesenbergii*, *M. flos-aquae*, and *Ap. flos-aquae* were even better than that of CIA. Some synthesized CIA esters, e.g., compounds **16**–**19**, **21,** and **24**, showed a relative selectivity in cyanobacteria inhibition. For instance, compound **24** showed excellent inhibition activities against *M. aeruginosa* and *Ap. flos-aquae* with EC_50_ of 1.38–5.29 µmol/L; however, its inhibition activities against other cyanobacteria species were moderate with EC_50_ of 26.58–(>100) µmol/L. Compounds **17**–**19**, and **21** showed excellent inhibition activities against *Ap. flos-aquae* with EC_50_ of 0.63–6.06 µmol/L; however, their inhibition activities against other cyanobacteria species were moderate with EC_50_ of 11.69–81.57 µmol/L. Compound **16** showed excellent inhibition activities against *M. wesenbergii* and *Ap. flos-aquae* with EC_50_ of 5.56–5.59 µmol/L; however, its inhibition activities against other cyanobacteria species were moderate with EC_50_ of 10.72–39.9 µmol/L.

The algicidal activities of HNA esters presented a strong specificity. Most HNA esters including compounds **27** (EC_50_ = 8.48 µmol/L), **29** (EC_50_ = 9.93 µmol/L), **30** (EC_50_ = 2.56 µmol/L), and **31** (EC_50_ = 5.94 µmol/L) exhibited excellent inhibition activities against *Ap. flos-aquae*, which are much better than HNA (EC_50_ = 66.58 µmol/L). However, their algicidal activities were much lower against other cyanobacteria species. The scatter plot of PCoA showed these HHA esters were assembled on the right side (Figure 1A), implying the large loading value on the first axis might have a selectivity for the algicidal activity.

Typically, the CA esters and amides did not achieve good algicidal effects. Only two CA amides, compounds 6 (EC_50_ = 7.27 µmol/L) and 7 (EC_50_ = 3.59 µmol/L), exhibited good inhibition activities against *M. flos-aquae*, and their activities were comparable to CA’s.

In terms of cyanobacteria species, *An. flos-aquae* showed a strong recalcitrance to these synthesized compounds. Only CIA esters and some HNA esters (compounds 29 and 30) exhibited a moderate algicidal activity on *An. flos-aquae*. The highest algicidal activity against *An. flos-aquae* was achieved by compound 19 with an EC_50_ of 30.27 µmol/L. In the scatter plot of PCoA, *An. flos-aquae* was significantly alienated from other cyanobacterial species (Figure 1B), suggesting its resistance to the inhibition effects of the synthesized compounds.

### 3.2. Structure–Activity Relationship Analysis

CIA series were the most active compounds with a broad-spectrum algicidal activity among these synthesized compounds. Compound **18** exhibited the best inhibition activities on four cyanobacteria species, and a moderate activity on *An. flos-aquae*. Its inhibitory activity on *Ap. flos-aquae* even reached 0.63 µmol/L, which is far stronger than the positive control CIA (EC_50_ = 26.1 µmol/L). Compound **18** was synthesized by CIA and naphthol. However, the activity of compound **22** containing a benzene ring was much lower than compound **18**, suggesting that the naphthalene ring might provide a higher binding ability to the target than the benzene ring. Moreover, the activity of compound **18** was also slightly stronger than that of other CIA fatty alcohol esters (compounds **15**–**17**, **19**–**21**, **23**, and **24**), demonstrating the importance of the substituent of the naphthalene ring.

Further, we analyzed the activities of CIA fatty alcohol esters against these five cyanobacteria species and the structure–activity relationship seemed to be contradictive on different species. The compounds with branched chains (**15**, **19**, and **21**) had lower activities against *M. aeruginosa*, while compounds with straight chains (**17**, **20**, **23**, and **24**) and ring structure (**16**) had better activities. Among them, compound **24** with the best activity was n-octyl cinnamate, which had the longest chain structure. The inhibition activity test on *M. wesenbergii* showed that the most active compound was cyclohexyl cinnamate (compound **16**), and there was no significant difference in activities between compounds with branched and straight chains. The compounds’ inhibition against *M. flos-aquae* showed that compounds **15**, **19**, and **21** with branched chains were slightly more active than those with straight chains (**17**, **20**, **23**, and **24**) and ring structure (**16**). After analysis of the compounds’ inhibition activities against *Ap. flos-aquae*, it was shown that there was no significant difference between compounds with branched and straight chains. In addition, the algicidal assays on *An. flos-aquae* found that compounds **15** and **19** with branched chains showed better activities than other compounds.

Compared with CIA esters, CA esters have *meta-* and *para-* hydroxyl substituents in the benzene ring; however, the activities of CA esters were greatly reduced. The possible reason is that hydroxyl substituents attenuate the aromaticity and steric structure of the benzene ring, thus affecting the binding ability of the compound to the target. The algicidal activities of HNA series compounds were stronger than the CA series but weaker than the CIA series, which may be related to the fact that the aromaticity of the naphthalene ring is slightly lower than that of the benzene ring.

Although CIA, CA, and other phenolic acids have been reported as allelochemicals to inhibit cyanobacteria growth, this is the first report that their ester and amide derivatives have inhibitory activities on the growth of cyanobacteria. By analyzing the data of this study, it can be found that compounds with some structures have the potential to be developed as algicides. Among these three series of ester derivatives, compounds **5**, **16**, and **30** with cyclohexyl ester substituents exhibited superior algicidal activities. It is worth mentioning that the inhibitory activities of these three compounds on *Ap. flos-aquae* are the best, which means they have potential to be developed as selective algaecides against *Ap. flos-aquae*. The steric hindrance near the ester group may be helpful to improve the activities of these compounds.

Steric hindrance can also enhance the activities of other compounds with large substituents. Octyl cinnamate (**24**) and octyl 3-hydroxy-2-naphthoate (**31**) exhibited good inhibitory activities on *M. aeruginosa* and *Ap. flos-aquae*. The EC_50_ value of compound **24** on *M. aeruginosa* reached 1.38 µmol/L, and it has the potential to be a selective algicide against *M. aeruginosa*.

### 3.3. 3D-QSAR Analysis

In order to further explore the correlation between the structure and activity of the compounds, and to provide guidance for the development of more potent algicides, a series of 3D-QSAR models were established based on the algicidal activities of the synthesized compounds in this study.

Before establishing the 3D-QSAR models, benzyl was chosen as a substructure to align the structure of the compounds. In the modelling, seven compounds were selected as the test set, while the rest of the compounds were used as the training set. The operation process mainly referred to the previous literature [22].

**Figure 2:**
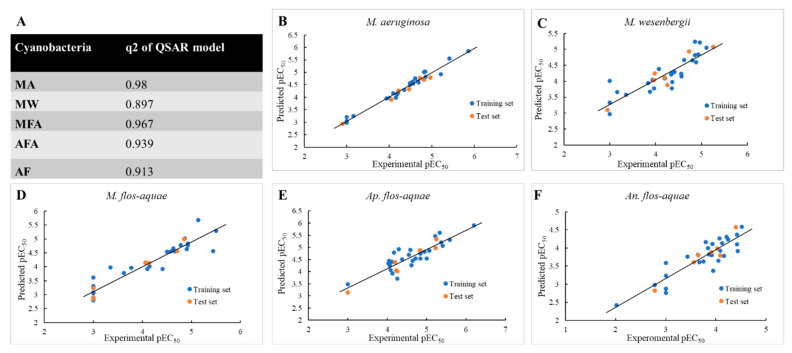
3D-QSAR analysis results of compounds’ inhibitory effects on cyanobacteria. (**A**): *q*^2^ values of all five 3D-QSAR models. (**B**–**F**): Plots of the predicted and experimental pEC_50_ values of the training and test sets.

As shown in Figure 2A, the cross-validation correlation coefficient (*q*^2^) values of the five constructed models were between 0.9 and 1, which proves that these models are credible [23]. The predicted and experimental pEC_50_ values of the 31 compounds are listed in the Supplementary Materials Table S1. Plots of the experimental pEC_50_ versus the predicted values are provided in Figure 2B–F.

**Figure 3:**
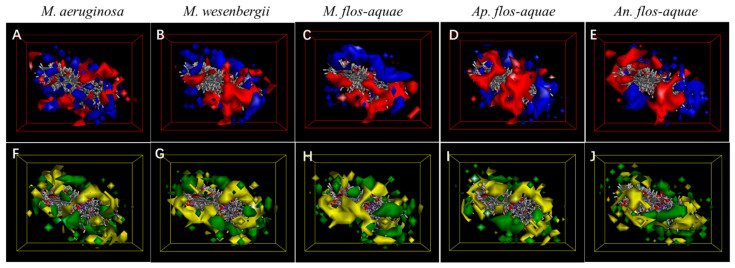
(**A**–**E**) 3D-QSAR model coefficients on electrostatic potential grids. Blue represents positive coefficients, and red represents negative coefficients. (**F**–**J**) 3D-QSAR model coefficients on van der Waals grids. Green represents positive coefficients, and yellow represents negative coefficients.

Moreover, the molecules aligned with the iso-surfaces of the 3D-QSAR model coefficients on electrostatic potential grids (Figure 3A–E) and van der Waals grids (Figure 3F–J) are shown. As described previously, the electrostatic map indicated red contours around regions where high electron density (negative charge) was expected to increase the activity, and blue contours represented areas where low electron density (partial positive charge) was expected to increase the activity [24]. The steric map derived from van der Waals analysis indicated areas where steric bulk was predicted to increase (green) or decrease (yellow) the activity [24].

For the models of *M. aeruginosa*, *M. wesenbergii*, and *M. flos-aquae*, the benzene ring from the acid group was mainly occupied by blue (Figure 3A–E), indicating that the low electron density would increase the activity of compounds, which is consistent with the previous activity evaluation result, i.e., when there were substituent groups with high electron density on the benzene ring, such as the hydroxyl substituent, or when the benzene ring was replaced by the naphthalene ring, the activity was affected. However, for the models of *Ap. flos-aquae* and *An. flos-aquae*, the benzene ring of the compounds was mainly occupied by red, and the high electron density would be beneficial for enhancing the activity. The algicidal activity evaluation results also showed that the naphthalene ring in the HNA ester compounds could enhance the activity against *Ap. flos-aquae* or *An. flos-aquae*.

As shown in Figure 3F–J, the compounds with small substituent groups in the benzene ring from the acid group exhibited better activities against all five cyanobacteria species, which confirmed that the hydroxyl substituents in the benzene ring influenced the activity of the compounds. Moreover, the naphthyl ring also influenced the activity. For the substituents, the corresponding models of different cyanobacteria species were different, but it was clear that for the compounds with aromatic substituents, yellow occupied the main position, indicating that the large substituents weaken the activity. Meanwhile for the fatty alcohol ester substituents, green occupied the main position, suggesting that big substituents could enhance the activity. In the previous structure–activity relationship analysis (Section 3.2), we mentioned that the steric hindrance of fatty alcohol chains could enhance the activity, while the benzene substituents, such as nitrobenzene, could seriously affect the activity. This is consistent with the results of QSAR analysis.

### 3.4. Physiological Assays

In order to further explore the inhibition mechanism of the synthesized compounds on cyanobacteria, we investigated the possible mechanisms involved in the inhibition process, including superoxide dismutase (SOD) activity, soluble protein content, and membrane lipid peroxidation. Compound **24** with the best algicidal activity on *M. aeruginosa* was chosen as an example and CIA was used as the control.

**Figure 4:**
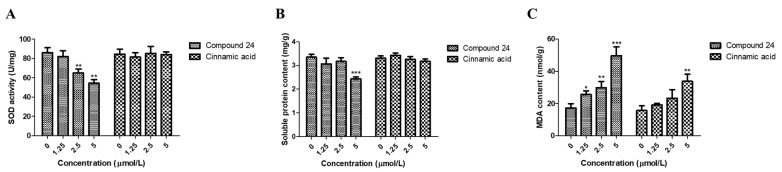
Superoxide dismutase (SOD) activity (**A**), and contents of soluble protein (**B**) and malondialdehyde (MDA) (**C**) in *M. aeruginosa* after 72-h exposure to compound **24** and CIA (0, 1.25, 2.5, and 5 µmol/L). Data are presented as mean values ± standard deviation (n = 3). * *p* < 0.05, ** *p* < 0.01, *** *p* < 0.001 vs. 0 µmol/L.

Under conditions of oxidative stress, SOD acts as an endogenous cellular defense barrier that degrades superoxide (O_2_^●−^) into oxygen and hydrogen peroxide, with the latter being further decomposed by glutathione peroxidase or catalase [25]. After treating *M. aeruginosa* with compound **24** and CIA for 72 h, the cellular SOD activity was measured, and the results are shown in Figure 4A. It can be seen that after exposure to compound **24** of 5 µmol/L, the SOD activity in *M. aeruginosa* decreased by 36.68%, whereas the activity of SOD did not change significantly after exposure of CIA of 5 µmol/L. This result indicated that compound **24** could influence the antioxidant defense system in *M. aeruginosa*, which might be related to the inhibition of the growth of *M. aeruginosa*. CIA had little effect on SOD activity at this concentration, suggesting that suppressing the SOD activity might not be the main mechanism of CIA inhibition on *M. aeruginosa*.

The changes of soluble proteins in *M. aeruginosa* in the exposure experiment are shown in Figure 4B. The soluble protein content in *M. aeruginosa* did not change significantly after treatment with CIA for 72 h. After the 72-h treatment of compound **24** on *M. aeruginosa*, only when the concentration increased to 5 µmol/L did the soluble protein content decrease by 27.46%. This result indicated that the main mechanism of compound **24** and CIA inhibiting the growth of *M. aeruginosa* was not through hindering of the synthesis of soluble proteins.

Malondialdehyde (MDA) is the product of membrane lipid peroxidation. The accumulated MDA can damage the membrane structure and function, increase membrane permeability, make cell metabolism imbalanced, and destroy the progress of various reactions in cells, even leading to cell death in severe cases [26]. In this experiment, it was found that when the concentrations of CIA and compound **24** were higher than 1.25 µmol/L, the MDA content of *M. aeruginosa* gradually increased. When the concentration of compound **24** was 1.25 µmol/L, the content of MDA increased significantly (*p* < 0.05). The change trends of MDA in the CIA-treated group and compound **24**-treated group were basically the same. This result indicated that CIA and compound **24** could significantly promote cell membrane lipid peroxidation, cause the accumulation of MDA, and finally inhibit the growth of *M. aeruginosa*.

## 4. Discussion

Due to the frequent outbreaks of cyanobacteria bloom, research on novel algicides has attracted more and more attention. At present, the reported algicides mainly focus on allelochemicals. In a study on *M. aeruginosa* inhibitors, researchers found that some plant extracts had good inhibitory effects, such as rice straw extract, *L. camara* leave extracts, etc. [27,28,29]. Further studies found that some allelochemicals with a single component also had inhibitory effects, such as gramine, cinnamic acid, artemisinin, etc. [9,30]. However, few articles on structural modification of these allelochemicals have been published. In this study, phenolic acid derivatives were innovatively synthesized as potential algicides, and lead compounds with excellent activity were found. Taking the inhibitors on *M. aeruginosa* for example, the EC_50_ of the best active compound **24** reached 1.38 µM (0.36 mg/L), while the EC_50_ values of previously reported allelochemicals have been basically at the mg/L level [9,31,32]. This result indicates that the algicides reported in this study are more efficient in inhibiting cyanobacteria with a lower concentration than most previously reported allelochemicals.

As part of the aquatic ecosystem, cyanobacteria are closely related to other organisms in the water, such as aquatic plants, fish, and zooplankton. Therefore, it is very important for the use of algicides that they just kill cyanobacteria without harming other species. Meanwhile, there are many kinds of cyanobacteria species in the water, and not all cyanobacteria species will cause water bloom. In the famous reported cyanobacteria bloom events in history, the dominant cyanobacteria species are also different. For example, in the Darling River cyanobacteria bloom event in 1991, the dominant cyanobacteria species was *Anabaena* [33]. In the Taihu Lake cyanobacteria bloom event in 2007, the dominant cyanobacteria species were *M. aeruginosa* and *M. wesenbergii* [16,34]. In the Dianchi Lake cyanobacteria bloom in 2007, the dominant cyanobacteria species were *M. aeruginosa* and *Ap. flos-aquae* [35]. Therefore, it is very important to employ selective algicides to control specific cyanobacteria bloom.

In this study, we found that CIA esters could inhibit the growth of water bloom-forming cyanobacteria, and some compounds had strong selectivity. For instance, the EC_50_ of compound **18** to *Ap. flos-aquae* was as low as 0.63 µM, while its activities against other cyanobacteria species were greater than 10 µM. The EC_50_ of compound **24** on *M. aeruginosa* was as low as 1.38 µM, while its activities on *M. wesenbergii* (EC_50_ = 38.62 µM) and *Microcystis flos-aquae* (EC_50_ = 26.58 µM) were much lower. These results indicated that these compounds could still inhibit cyanobacteria growth at low concentrations. Another important finding of this study is that the selective inhibition of naphthoic acid ester derivatives on *Ap. flos-aquae* was generally stronger than that on other cyanobacteria species, making us strongly interested in this compound skeleton. The mechanism underlying the inhibition effect is worthy of further investigation. From the environmentally friendly aspect, it was necessary to find out selective algicides without harming other species. In this study, we just validated the selectivity of algicides on different cyanobacteria. The effects of these algicides on other species, like microalgae and zooplankton, need to be clarified in the future.

With the development of drug discovery, more and more new types of algicides have emerged, such as metal organic chelates, nanomaterials, etc. [36,37]. Compared with these new algicides, the allelochemical relatives reported in this study may be more environmentally friendly with a lower cost and higher application value.

## 5. Conclusions

In general, the synthesized compounds especially CIA esters, exhibited excellent inhibitory activities against multiple cyanobacteria species except *An. flos-aquae*. Although some of the other series of compounds except CIA esters were not as active as the controls, all of the synthesized compounds showed obvious advantages, including easy synthesis and low cost. Structure–activity analysis and 3D-QSAR modelling demonstrated that steric hindrance could enhance the activity of CIA esters, and provided theoretical guidance for prediction of the activities and further structure optimization of the compounds. The physiological assays preliminarily revealed that the possible action mechanism of CIA esters might involve cell membrane lipid peroxidation. Therefore, CIA esters as a new type of algal inhibitors have great potential as lead compounds for the development of environmentally friendly algicides. This result has provided a direction for further design and optimization of the chemical structure of potential algicides.

## Figures and Tables

**Table 1:** Algicidal activities of the synthesized compounds **1**–**31** and parent compounds.

Compounds		EC_50_ (µmol/L)				
		*M. aeruginosa*	*M. wesenbergii*	*M. flos-aquae*	*Ap. flos-aquae*	*An. flos-aquae*
CA esters	**1**	>100	>100	>100	>100	>100
	**2**	>100	>100	>100	>100	>100
	**3**	61.52	65.00	73.40	55.01	>100
	**4**	32.50	24.10	23.20	75.15	>100
	**5**	27.40	64.90	31.50	24.10	>100
CA amides	**6**	27.54	10.82	7.27	54.80	>150
	**7**	70.38	27.24	3.59	85.30	69.59
	**8**	>100	>100	80.00	28.00	>150
	**9**	>100	>100	>100	50.56	75.63
	**10**	89.30	>100	87.00	58.80	93.88
	**11**	>100	62.50	>100	42.10	>150
	**12**	115.40	109.60	>100	19.10	121.4
CIA esters	**13**	65.54	>100	>100	92.78	88.81
	**14**	99.60	43.42	38.60	14.49	>100
	**15**	20.55	7.73	13.70	22.72	36.89
	**16**	10.72	5.56	19.61	5.59	39.9
	**17**	15.26	11.69	16.47	3.94	81.57
	**18**	13.93	13.17	12.56	0.63	61.15
	**19**	20.81	13.81	11.78	4.59	30.27
	**20**	18.98	18.82	14.15	14.84	82.09
	**21**	24.51	13.88	11.77	6.06	57.33
	**22**	80.72	>100	71.31	89.85	>100
	**23**	15.29	27.81	21.66	14.36	>100
	**24**	1.38	38.62	26.58	5.29	>100
HNA esters	**25**	33.84	105.60	>100	62.45	>100
	**26**	32.34	46.26	>100	11.39	>100
	**27**	24.47	134.68	>100	8.48	>100
	**28**	61.62	>100	>100	81.31	>100
	**29**	28.35	44.44	>100	9.93	37.99
	**30**	14.34	15.88	>100	2.56	38.23
	**31**	35.50	55.10	>100	5.94	224.24
Controls	CA	6.18	44.00	3.13	74.00	138.00
	CIA	3.87	85.88	169.92	26.10	>200
	HNA	43.84	117.18	>100	66.58	84.64

## Data Availability

Data are contained within the article, and materials can be requested from the authors upon reasonable request.

## Supplementary Materials

The following are available online at https://www.mdpi.com/article/10.3390/biology10111145/s1, Supplementary Materials S1: The characterization data of compounds **1**–**31**, Table S1: Predicted and experimental pEC_50_ values of 3D-QSAR models.

## References

[1] Cai W.-J., Hu X., Huang W.-J., Murrell M., Lehrter J.C., Lohrenz S., Chou W.-C., Zhai W.-D., Hollibaugh J.T., Wang Y. (2011). Acidification of subsurface coastal waters enhanced by eutrophication. Nat. Geosci..

[2] Zhu Y., Ling J., Li L., Guan X. (2020). The effectiveness of bisulfite-activated permanganate technology to enhance the coagulation efficiency of *Microcystis aeruginosa*. Chin. Chem. Lett..

[3] Fu J. (2016). Issue of Cyanobacteria Blooms in Taihu Lake, China. J. Environ. Sci. Manag..

[4] Hernandez B.Y., Zhu X., Sotto P., Paulino Y. (2021). Oral exposure to environmental cyanobacteria toxins: Implications for cancer risk. Environ. Int..

[5] Xiong J., Xie R., Zhang H., Gao J., Wang J., Liang Q. (2021). Nitrite-responsive hydrogel for long-term and smart control of cyanobacteria bloom. J. Hazard. Mater..

[6] Leao P.N., Pereira A.R., Liu W.-T., Ng J., Pevzner P.A., Dorrestein P.C., Konig G.M., Vasconcelos V.M., Gerwick W.H. (2010). Synergistic allelochemicals from a freshwater cyanobacterium. Proc. Natl. Acad. Sci. USA.

[7] Zhang X.-W., Fu J., Song S., Zhang P., Yang X.-H., Zhang L.-R., Luo Y., Liu C.-H., Zhu H.-L. (2014). Interspecific Competition between *Microcystis aeruginosa* and *Anabaena flos-aquae* from Taihu Lake, China. Z. Naturforsch. C.

[8] Nakai S., Inoue Y., Hosomi M. (2001). Algal growth inhibition effects and inducement modes by plant-producing phenols. Water Res..

[9] Ni L., Acharya K., Hao X., Li S. (2012). Isolation and identification of an anti-algal compound from *Artemisia annua* and mechanisms of inhibitory effect on algae. Chemosphere.

[10] Gao Y.N., Liu B.Y., Ge F.J., He Y., Lu Z.Y., Zhou Q.H., Zhang Y.Y., Wu Z.B., Gao Y.N., Liu B.Y. (2015). Joint effects of allelochemical nonanoic acid, N-phenyl-1-naphtylamine and caffeic acid on the growth of *Microcystis aeruginosa*. Allelopath. J..

[11] He M., Zhang T., Wu A., Nie L. (2008). Inhibition of Cinnamic acid on *Microcystis aeruginosa* K. and *Scenedesmus arcuatus* L.. Chin. J. Appl. Environ. Biol..

[12] Gao Y.N., Liu B.Y., Xu D., Zhou Q.H., Hu C.Y., Ge F.J., Zhang L.P., Wu Z.B. (2011). Phenolic Compounds Exuded from Two Submerged Freshwater Macrophytes and Their Allelopathic Effects on *Microcystis aeruginosa*. Pol. J. Environ. Stud..

[13] Zhu B., Jun H., Ting S., Wei W., Junyi Z. Study on the species and succession of cyanobacteria bloom in Taihu Lake. Proceedings of the 2014 Annual Conference of the Chinese Society for Environmental Science (Chapter 5).

[14] Tan X., Kong F., Zeng Q., Cao H., Qian S., Zhang M. (2009). Seasonal variation of Microcystis in Lake Taihu and its relationships with environmental factors. J. Environ. Sci..

[15] Yamamoto Y., Nakahara H. (2005). The formation and degradation of cyanobacterium *Aphanizomenon flos-aquae* blooms: The importance of pH, water temperature, and day length. Limnology.

[16] Xu Y., Wu Z., Yu B., Peng X., Yu G., Wei Z., Wang G., Li R. (2008). Non-microcystin producing *Microcystis wesenbergii* (Komárek) Komárek (Cyanobacteria) representing a main waterbloom-forming species in Chinese waters. Environ. Pollut..

[17] Wan J., Guo P., Zhang S. (2014). Response of the cyanobacterium *Microcystis flos-aquae* to levofloxacin. Environ. Sci. Pollut. Res..

[18] Peng Y., Zhang Z., Wang M., Shi X., Zhou Y., Zhou Y., Kong Y. (2020). Inactivation of harmful *Anabaena flos-aquae* by ultrasound irradiation: Cell disruption mechanism and enhanced coagulation. Ultrason. Sonochemistry.

[19] Pierce J., Suelter C. (1977). An evaluation of the Coomassie brilliant blue G-250 dye-binding method for quantitative protein determination. Anal. Biochem..

[20] Yong Z., Tang H.R., Luo Y. (2008). Variation in Antioxidant Enzyme Activities of Two Strawberry Cultivars with Short-term Low Temperature Stress. World J. Agric. Sci..

[21] Heath R. (1968). Photoperoxidation in isolated chloroplasts: I. Kinetics and stoichiometry of fatty acid peroxidation. Arch. Biochem. Biophys.

[22] Luo Y., Zhou Y., Fu J., Zhu H.-L. (2014). 4,5-Dihydropyrazole derivatives containing oxygen-bearing heterocycles as potential telomerase inhibitors with anticancer activity. RSC Adv..

[23] Liu S., Luo Y., Fu J., Zhou J., Kyzas G. (2016). Molecular docking and 3D-QSAR studies on the glucocorticoid receptor antagonistic activity of hydroxylated polychlorinated biphenyls. SAR QSAR Environ. Res..

[24] Luo Y., Zhou Y., Song Y., Chen G., Wang Y.-X., Tian Y., Fan W.-W., Yang Y.-S., Cheng T., Zhu H.-L. (2018). Optimization of substituted cinnamic acyl sulfonamide derivatives as tubulin polymerization inhibitors with anticancer activity. Bioorganic Med. Chem. Lett..

[25] Yasui K., Baba A. (2006). Therapeutic potential of superoxide dismutase (SOD) for resolution of inflammation. Inflamm. Res..

[26] Michael P.I., Krishnaswamy M. (2011). The effect of zinc stress combined with high irradiance stress on membrane damage and antioxidative response in bean seedlings. Environ. Exp. Bot..

[27] Kong C.H., Wang P., Zhang C.X., Zhang M.X., Hu F. (2006). Herbicidal potential of allelochemicals from *Lantana camara* against *Eichhornia crassipes* and the alga *Microcystis aeruginosa*. Weed Res..

[28] Park M.-H., Han M.-S., Ahn C.-Y., Kim H.-S., Yoon B.-D., Oh H.-M. (2006). Growth inhibition of bloom-forming cyanobacterium *Microcystis aeruginosa* by rice straw extract. Lett. Appl. Microbiol..

[29] Zhao W., Zheng Z., Zhang J., Roger S.-F., Luo X. (2019). Allelopathically inhibitory effects of eucalyptus extracts on the growth of *Microcystis aeruginosa*. Chemosphere.

[30] Hong Y., Hu H.-Y., Xie X., Sakoda A., Sagehashi M., Li F.-M. (2009). Gramine-induced growth inhibition, oxidative damage and antioxidant responses in freshwater cyanobacterium *Microcystis aeruginosa*. Aquat. Toxicol..

[31] Hou X., Huang J., Tang J., Wang N., Zhang L., Gu L., Sun Y., Yang Z., Huang Y. (2019). Allelopathic inhibition of juglone (5-hydroxy-1,4-naphthoquinone) on the growth and physiological performance in *Microcystis aeruginosa*. J. Environ. Manag..

[32] Yi Y.-L., Yu X.-B., Zhang C., Wang G.-X. (2015). Growth inhibition and microcystin degradation effects of *Acinetobacter guillouiae* A2 on *Microcystis aeruginosa*. Res. Microbiol..

[33] Bowling L., Baker P. (1996). Major cyanobacterial bloom in the Barwon-Darling River, Australia, in 1991, and underlying limnological conditions. Mar. Freshw. Res..

[34] Li M., Xiao M. (2016). Environmental factors related to the dominance of *Microcystis wesenbergii* and *Microcystis aeruginosa* in a eutrophic lake. Environ. Earth Sci..

[35] Xing W., Huang W.-M., Liu Y.-D., Li D., Shen Y.-W., Li G.-B. (2007). Environmental mechanism of change in cyanobacterial species composition in the northeastern part of Lake Dianchi (China). Fresen. Env. Bull..

[36] Fan G., Zhou J., Zheng X., Chen W. (2018). Growth Inhibition of *Microcystis aeruginosa* by Copper-based MOFs: Performance and Physiological Effect on Algal Cells. Appl. Organomet. Chem..

[37] Sankar R., Prasath B.B., Nandakumar R., Santhanam P., Shivashangari K.S., Ravikumar V. (2014). Growth inhibition of bloom forming cyanobacterium *Microcystis aeruginosa* by green route fabricated copper oxide nanoparticles. Environ. Sci. Pollut. Res..

