# Supplementary material for: Novel Algicides against Bloom-Forming Cyanobacteria from Allelochemicals: Design, Synthesis, Bioassay, and 3D-QSAR Study
Source: Biology (Basel). 2021 Nov 6;10(11):1145. doi: 10.3390/biology10111145 (PMC8614697; doi:10.3390/biology10111145)
Supplement: Supplementary file 1 [file biology-10-01145-s001.zip › biology-1420984-supplementary.pdf]

Supplementary Materials

Supplementary Materials S1. The characterization data of compounds **1-31**.

(E)-4-Nitrophenyl 3-(3,4-dihydroxyphenyl)acrylate (1)

Yellow crystals. Yield: 62%. Mp: 202-203 ºC. ESI MS: 324.0 $[M + Na]^+$. ^1H NMR (300 MHz, DMSO-d_6, δ ppm): 9.74 (s, 1H), 9.19 (s, 1H), 8.32 (d, 2H, J = 9.0 Hz), 7.75 (d, 1H, J = 15.9 Hz), 7.51 (d, 2H, J = 9.2 Hz), 7.16 (d, 1H, J = 1.9 Hz), 7.13 (dd, 1H, J1 = 8.2 Hz, J2 = 1.9 Hz), 6.81 (d, 1H, J = 8.2 Hz), 6.52 (d, 1H, J = 15.7 Hz). Anal. calc. for $C_{15}H_{11}NO_6$: C, 59.80%; H, 3.68%; N, 4.65%. Found: C, 59.64%; H, 3.49%, N, 4.87%.

(E)-2,4,6-Trinitrophenyl 3-(3,4-dihydroxyphenyl)acrylate (2)

Yellow crystals. Yield: 68%. Mp: 204-205 ºC. ESI MS: 414.0 $[M + Na]^+$. ^1H NMR (300 MHz, DMSO-d_6, δ ppm): 9.81 (s, 1H), 9.23 (s, 1H), 9.15 (s, 2H), 7.76 (d, 1H, J = 15.9 Hz), 7.16 (d, 1H, J = 1.9 Hz), 7.11 (dd, 1H, J1 = 8.2 Hz, J2 = 1.9 Hz), 6.83 (d, 1H, J = 8.2 Hz), 6.53 (d, 1H, J = 15.9 Hz). Anal. calc. for $C_{15}H_9N_3O_{10}$: C, 46.05%; H, 2.32%; N, 10.74%. Found: C, 46.24%; H, 2.43%, N, 10.62%.

(E)-Phenethyl 3-(3,4-dihydroxyphenyl)acrylate (3)

White crystals. Yield: 58%. Mp: 126-127 ºC. ESI MS: 307.0 $[M + Na]^+$. ^1H NMR (300 MHz, $CDCl_3$, δ ppm): 7.56 (d, 1H, J = 15.9 Hz), 7.44-7.34 (m, 5H), 7.11 (d, 1H, J = 2.2 Hz), 7.04 (dd, 1H, J1 = 8.0 Hz, J2 = 2.2 Hz), 6.80 (d, 1H, J = 8.0 Hz), 6.33 (d, 1H, J = 15.9 Hz), 4.40 (t, 2H, J = 6.8 Hz), 2.90 (t, 2H, J = 6.8 Hz). Anal. calc. for $C_{17}H_{16}O_4$: C, 71.82%; H, 5.67%. Found: C, 71.67%; H, 5.53%. The spectral data agreed with previously reported data [1].

Ethyl (E)-3-(3,4-dihydroxyphenyl)acrylate (4)

Yellow powder. Yield 55%, Mp: 146-147 ºC. ^{1}H-NMR (300 MHz, $CDCl_3$, δ ppm): 1.3076-1.3551 (m, 3H), 4.2203-4.2916 (q, J = 7.14 Hz, 2H), 6.2234-6.2764 (d, J=15.9Hz, 1H), 6.8214-6.9165 (m, 1H), 6.9708-7.0043 (dd, J1=1.83, J2=8.22Hz, 1H), 7.0811-7.0872 (d, J=1.83Hz, 1H), 7.5481-7.6017 (d, J=16.08, 1H). MS (ESI): 209.07 ($[M + H]^+$). Anal. calc. for $C_{11}H_{12}O_4$: C, 63.45; H, 5.81%; found: C, 63.23; H, 5.79%. The spectral data agreed with previously reported data [2].

Cyclohexyl (E)-3-(3,4-dihydroxyphenyl)acrylate (5)

Yellow oil. Yield 53%, Mp: 149-150 ºC. ^{1}H-NMR (300 MHz, $CDCl_3$, δ ppm): 1.0680-1.1796 (m, 10H), 4.7476-4.8067(m, 1H), 6.1155-6.1685 (s, J=15.9Hz, 1H), 6.7525-6.7800 (s, J=8.25Hz, 1H), 6.8604-6.8330 (s, J=8.1Hz, 1H), 7.0159-7.0208 (s, J=1.47Hz, 1H), 7.3999-7.4322 (s, J=9.69Hz, 1H). MS (ESI): 263.12 ($[M + H]^+$). Anal. calc. for $C_{15}H_{18}O_4$: C, 68.69; H, 6.92%; found: C, 68.34; H, 6.99%.

(E)-3-(3,4-Dihydroxyphenyl)-N,N-diisopropylacrylamide (6)

Yellow crystal. Yield: 55%. Mp: 164-166 ºC. ESI MS: 264.16 $[M + Na]^+$. ^{1}H NMR (300 MHz, $CDCl_3$, δ ppm): 1.3574 (d, J=21.98Hz, 12H), 3.9735 (m, 2H), 6.6785 (d, J=15.13Hz, 1H), 6.8507 (d, J=9.12Hz, 1H), 7.0293 (dd, J1=1.83, J2=8.19Hz, 2H), 7.3643 (d, J=15.12, 1H), 9.1982 (s, 2H). Anal. calc. for $C_{15}H_{21}NO_3$: C, 68.42%; H, 8.04%, found: C, 68.58%; H, 8.25%.

(E)-N-Benzyl-3-(3,4-dihydroxyphenyl)acrylamide (7)

Colorless crystal. Yield: 65%. Mp: 235-237 ºC. ESI MS: 270.11 $[M + Na]^+$. ^1H NMR (300 MHz, $CDCl_3$, δ ppm): 4.62 (s, 2H), 6.5384 (d, J=15.16Hz, 1H), 6.7095 (d, J=8.09, 1H), 6.9108 (d, J=8.09Hz, 1H), 7.0133 (s, 1H), 7.1324(d, J=8.09Hz, 1H), 7.2831-7.2915 (m, 5H), 9.4305 (s, 1H), 9.4714 (s, 1H), 9.8093 (s, 1H). Anal. calc. for $C_{16}H_{15}NO_3$: C, 71.36%; H, 5.61%. Found: C, 71.19%; H, 5.38%.

(E)-3-(3,4-Dihydroxyphenyl)-N,N-dipropylacrylamide (8)

Light yellow powder. Mp: 212-213 ºC. ^1H NMR (300 MHz, DMSO-d_6, δ ppm): 0.963 (t, J=4.5Hz, 6H), 1.631-1.672 (m, 4H), 3.3865 (t, J=7.25Hz, 4H), 6.6658 (d, J=15.08Hz, 1H), 6.8913 (d, J=9.01Hz, 1H), 6.9915 (dd, J1=1.82, J2=8.16Hz, 2H), 7.8216 (d, J=15.08, 1H). ESI MS: 264.16 $[M + Na]^+$. Anal. calc. for $C_{15}H_{21}NO_3$: C, 68.42%; H, 8.04%; found: C, 68.21%; H, 8.09%.

(E)-3-(3,4-Dihydroxyphenyl)-N-ethylacrylamide (9)

Light green crystal. Mp: 117-118 ºC. ^1H NMR (300 MHz, DMSO-d_6, δ ppm): 1.2418 (t, J=7.14Hz, 3H), 3.6899 (s, 1H), 4.1197-4.1910 (q, J=7.14Hz, 2H), 6.2295-6.2990 (dd, J1=4.95Hz, J2=15.9Hz, 1H), 6.7635 (d, J=8.04Hz, 1H), 6.9854-7.0604 (m, 2H), 7.4807 (dd, J1=4.95Hz, J2=16.11Hz, 1H), 9.3561 (s, 2H). ESI MS: 208.09 $[M + Na]^+$. Anal. calc. for $C_{11}H_{13}NO_3$: C, 63.76%; H, 6.32%, found: C, 63.68%; H, 6.67%.

(E)-3-(3,4-Dihydroxyphenyl)acrylamide hydrate (10)

Colorless crystal. Mp: 122-125 ºC. ^1H NMR (300 MHz, DMSO-d_6, δ ppm): 6.2264-6.2959 (dd, J1=4.95, J2=15.9Hz, 1H), 6.7489-6.7757 (d, J=8.04Hz, 1H), 6.9909-7.0506 (m, 2H), 7.4426-

7.5115 (J1=4.77Hz, J2=15.87Hz, 1H), 9.1336 (s, 1H), 9.5859 (s, 1H). ESI MS: 198.07 [M + Na]$^+$. Anal. calc. for $C_9H_{11}NO_4$: C, 54.82%; H, 5.62%, found: C, 54.68%; H, 5.67%.

(E)-3-(3,4-Dihydroxyphenyl)-N-(p-tolyl)acrylamide (11)

Colorless crystal. Mp: 199-200 ºC. ^{1}H NMR (300 MHz, DMSO-d$_6$, δ ppm): 2.2598 (s, 3H), 6.5279 (d, J=15.54Hz, 1H), 6.776 (d, J=8.22, 1H), 6.9 (d, J=8.07Hz, 1H), 7.0019 (s, 1H), 7.1201 (d, J=8.04Hz, 2H), 7.3820 (d, J=15.57Hz, 1H), 7.5679 (d, J=8.22, 1H), 9.1629 (s, 1H), 9.4208 (s, 1H), 9.9602 (s, 1H). ESI MS: 270.11 [M + Na]$^+$. Anal. calc. for $C_{16}H_{15}NO_3$: C, 71.36%; H, 5.61%, found: C, 71.49%; H, 5.82%.

(E)-N,N-Dibutyl-3-(3,4-dihydroxyphenyl)acrylamide (12)

White powder. Mp: 134-135 ºC. ^{1}H NMR (300 MHz, $CDCl_3$, δ ppm): 0.9205-0.9943 (q, J=7.32Hz, 6H), 1.2594-1.4320 (m, 4H), 1.5331-1.6746 (m, 4H), 3.3607-3.4558 (m, 4H), 6.6251-6.6763 (d, J=15.36Hz, 1H), 6.8671-6.8946 (d, J=8.25Hz, 1H), 6.9708-7.0043 (dd, J1=1.83, J2=8.22Hz, 2H), 7.6426-7.6938 (d, J=15.26, 1H). ESI MS: 292.19 [M + Na]$^+$. Anal. calc. for $C_{17}H_{25}NO_3$: C, 70.07%; H, 8.65%, found: C, 70.23%; H, 8.78%.

4-Nitrophenyl cinnamate (13)

Yellow powder. Mp: 225-227°C. ^{1}H NMR (300 MHz, $CDCl_3$, δ ppm): 6. 3504 (d, 1H, J = 15.08 Hz), 7.2517-7.2309 (m, 2H), 7.3409-7.3507 (m, 2H), 7.3802-7.4013 (m, 2H), 7.5316 (dd, 2H, J1 = 2.14 Hz, J2 = 8.06 Hz), 8.2503 (d, 2H, J = 16.09 Hz). ESI MS: 270.07 [M + Na]$^+$. Anal. calc. for $C_{15}H_{11}NO_4$: C, 66.91%; H, 4.12%, found: C, 66.83%; H, 4.07%.

3,4,5-Trinitrophenyl cinnamate (14)

Light brown powder. Mp: 253-254°C. ^{1}H NMR (300 MHz, $CDCl_3$, δ ppm): 6.4769 (d, J=15.18Hz, 1H), 7.4698-7.4794 (m, 2H), 7.6009-7.6308 (m, 2H), 7.9482-7.9907 (m, 2H), 8.5216 (s, 2H). ESI MS: 360.04 $[M + Na]^+$. Anal. calc. for $C_{15}H_9N_3O_8$: C, 50.15%; H, 2.53%, found: C, 50.02%; H, 2.40%.

tert-Butyl cinnamate (15)

White powder. Mp: 125-127°C. ^{1}H NMR (300 MHz, $CDCl_3$, δ ppm): 1.5327 (s, 12H), 6.3074 (d, J=16.16Hz, 1H), 7.3524-7.3804 (m, 3H), 7.4516 (d, J=15.19Hz, 1H), 7.6502-7.6716 (m, 2H). ESI MS: 205.12 $[M + Na]^+$. Anal. calc. for $C_{13}H_{16}O_2$: C, 76.44%; H, 7.90%, found: C, 76.23%; H, 7.98%.

Cyclohexyl cinnamate (16)

Light yellow crystal. Mp: 114-115°C. ^{1}H NMR (300 MHz, $CDCl_3$, δ ppm): 1.4036-1.5507 (m, 8H), 1.7093-1.9451(m, 2H), 4.5032-4.7704 (m, 1H), 6.2245 (d, J=15.16Hz, 1H), 7.3208-7.3515 (m, 3H), 7.4953-7.5307 (m, 3H). ESI MS: 232.14 $[M + Na]^+$. Anal. calc. for $C_{15}H_{18}O_2$: C, 78.23%; H, 7.88%, found: C, 78.15%; H, 7.69%.

Ethyl cinnamate (17)

White powder. Mp: 108-109°C. ^{1}H NMR (300 MHz, $CDCl_3$, δ ppm): 1.2394 (t, J=8.16Hz, 3H), 4.1284 (q, J=8.19Hz, 2H), 6.2409 (d, J=15.18Hz, 1H), 7.3284-7.3907 (m, 3H), 7.4735-7.5516(m, 3H). ESI MS: 177.09 $[M + Na]^+$. Anal. calc. for $C_{11}H_{12}O_2$: C, 74.98%; H, 6.86%, found: C, 74.88%; H, 6.81%.

Naphthalen-2-yl cinnamate (18)

White powder. Mp: 182-184°C. ^{1}H NMR (300 MHz, $CDCl_3$, δ ppm): 6.3318 (d, J=15.16Hz, 1H), 7.1456 (dd, J1=1.58Hz, J2=7.69Hz, 1H), 7.3374-7.5409 (m, 9H), 7.7833 (dd, J1=1.56Hz, J2=7.58Hz, 1H), 7.9103-7.9843 (m, 2H). ESI MS: 276.11 $[M + Na]^+$. Anal. calc. for $C_{19}H_{14}O_2$: C, 83.19%; H, 5.14%, found: C, 83.11%; H, 5.22%.

Isopropyl cinnamate (19)

Yellow crystal. Mp: 104-105°C. ^{1}H NMR (300 MHz, $CDCl_3$, δ ppm): 1.2089 (d, J=6.88Hz, 6H), 4.6912-4.9015 (m, 1H), 6.5438 (d, J=15.17Hz, 1H), 7.3305-7.3829 (m, 3H), 7.4908-7.5314 (m, 3H). ESI MS: 191.10 $[M + Na]^+$. Anal. calc. for $C_{12}H_{14}O_2$: C, 75.76%; H, 7.42%; found: C, 75.64%; H, 7.3%.

Butyl cinnamate (20)

White powder. Yield: 77%. Mp: 134-135 ºC. ESI MS: 227.1 $[M + Na]^+$. ^{1}H NMR (300 MHz, $CDCl_3$, δ ppm): 7.76 (d, 1H, J = 15.9 Hz), 7.54 (dd, 2H, J1 = 7.2 Hz, J2 = 2.3 Hz), 7.42-7.33 (m, 3H), 6.48 (d, 1H, J = 16.0 Hz), 4.14 (t, 2H, J = 6.7 Hz), 1.67 (m, 2H), 1.44 (m, 2H), 0.94 (t, 3H, J = 7.4 Hz). Anal. calc. for $C_{13}H_{16}O_2$: C, 76.44%; H, 7.90%. Found: C, 76.25%; H, 7.98%. The spectral data agreed with previously reported data [1].

Isobutyl cinnamate (21)

Light yellow crystal. Yield: 65%. Mp: 129-130°C. ^{1}H NMR (300 MHz, $CDCl_3$, δ ppm): 0.7665 (d, J=6.84Hz, 6H), 1.7109-1.9265 (m, 1H), 3.7808 (d, J=6.92Hz, 1H), 6.3241 (d, J=15.18Hz,

1H), 7.3314-7.3984 (m, 3H), 7.4806-7.5652(m, 3H). ESI MS: 205.12 [M + Na]$^+$. Anal. calc. for $C_{13}H_{16}O_2$: C, 76.44%; H, 7.90%, found: C, 76.53%; H, 7.99%.

Phenethyl cinnamate (22)

Colorless crystals. Yield: 72%. Mp: 53-54 ºC. ESI MS: 275.1 [M + Na]$^+$. ^{1}H NMR (300 MHz, $CDCl_3$, δ ppm): 7.71 (d, 1H, J = 16.0 Hz), 7.52 (dd, 2H, J1 = 8.1 Hz, J2 = 2.2 Hz), 7.39-7.37 (m, 3H), 7.33 (m, 2H), 7.28-7.23 (m, 3H), 6.46 (d, 1H, J = 16.0 Hz), 4.45 (t, 2H, J = 7.0 Hz), 2.94 (t, 2H, J = 7.0 Hz). Anal. calc. for $C_{17}H_{16}O_2$: C, 80.93%; H, 6.39%. Found: C, 80.59%; H, 6.28%. The spectral data agreed with previously reported data [2].

Methyl cinnamate (23)

Light yellow crystal. Yield: 72%. Mp: 40-41°C. ^{1}H NMR (300 MHz, $CDCl_3$, δ ppm): 3.8102 (s, 3H), 6.3743 (d, J=15.08Hz, 1H), 7.3225-7.3893 (m, 3H), 7.5694-7.5899(m, 3H). ESI MS: 163.07 [M + Na]$^+$. Anal. calc. for $C_{10}H_{10}O_2$: C, 74.06%; H, 6.22%, found: C, 74.19%; H, 6.33%.

Octyl cinnamate (24)

Yellow crystal. Yield: 55%. Mp: 68-69°C. ^{1}H NMR (300 MHz, $CDCl_3$, δ ppm): 0.9318 (t, J=8.12Hz, 3H), 1.2597-1.3209 (m, 8H), 1.4211-1.4409 (m, 2H), 1.5531-1.6322(m, 2H), 3.8432 (t, J=7.12Hz, 2H), 6.2208 (d, J=15.16Hz, 1H), 7.3114-7.3503 (m, 3H), 7.5369-7.5671 (m, 3H). ESI MS: 261.18 [M + Na]$^+$. Anal. calc. for $C_{17}H_{24}O_2$: C, 78.42%; H, 9.29%, found: C, 78.19%; H, 9.22%.

2H-Naphtho[2,3-b]oxet-2-one (25)

Colorless oil. Yield: 32%. ^{1}H NMR (300 MHz, $CDCl_3$, δ ppm): 7.27 (s, 1H), 7.63-7.55 (m, 2H), 7.89 (dd, 1H, J1 = 8.2 Hz, J2 = 2.1 Hz), 8.02 (dd, 1H, J1 = 8.1 Hz, J2 = 2.1 Hz), 8.70 (s, 1H). ESI MS: 171.04 $[M + Na]^+$. Anal. calc. for $C_{11}H_6O_2$: C, 77.64%; H, 3.55%, found: C, 77.35%; H, 3.66%.

Methyl 3-hydroxy-2-naphthoate (26)

Colorless crystal. Yield: 42%. Mp: 165-167°C. ^{1}H NMR (300 MHz, $CDCl_3$, δ ppm): 4.1357 (s, 3H), 7.3503 (m, 1H), 7.4831 (m, 1H), 7.8605-7.8933 (m, 3H), 8.3041 (s, 1H), 12.0086 (s, 1H). ESI MS: 203.07 $[M + Na]^+$. Anal. calc. for $C_{12}H_{10}O_3$: C, 71.28%; H, 4.98%, found: C, 71.12%; H, 5.09%.

Phenethyl 3-hydroxy-2-naphthoate (27)

Yellow crystal. Yield: 52%. Mp: 251-253°C. ^{1}H NMR (300 MHz, $CDCl_3$, δ ppm): 3.169 (t, J=7.15Hz, 2H), 4.6987 (t, J=7.12Hz, 2H), 7.1265-7.1637 (m, 5H), 7.3517 (dd, J1=1.86, J2=7.81, 1H), 7.534 (dd, J1=1.82, J2=7.64, 1H), 7.8891-7.9174 (m, 3H), 8.2277 (s, 1H), 13.3135 (s, 1H). ESI MS: 293.11 $[M + Na]^+$. Anal. calc. for $C_{19}H_{16}O_3$: C, 78.06%; H, 5.52%, found: C, 78.24%; H, 5.45%.

Isopropyl 3-hydroxy-2-naphthoate (28)

Light yellow crystal. Mp: 171-172°C. ^{1}H NMR (300 MHz, $CDCl_3$, δ ppm): 1.4315 (d, J=6.92Hz, 6H), 5.298 (m, 1H), 7.3734 (dd, J1=1.6Hz, J2=7.67Hz, 1H), 7.5698 (dd, J1=1.64, J2=7.62, 1H). 7.8639-7.8974 (m, 3H), 8.3561 (s, 1H), 12.4375 (s, 1H). ESI MS: 231.10 $[M + Na]^+$. Anal. calc. for $C_{14}H_{14}O_3$: C, 73.03%; H, 6.13%, found: C, 73.14%; H, 6.08%.

Butyl 3-hydroxy-2-naphthoate (29)

Yellow oil. Yield: 62%. ESI MS: 267.0 $[M + Na]^+$. ^{1}H NMR (300 MHz, $CDCl_3$, δ ppm): 10.53 (s, 1H), 8.49 (s, 1H), 7.80 (dd, 1H, J1 = 8.0 Hz, J2 = 2.0 Hz), 7.68 (dd, 1H, J1 = 8.2 Hz, J2 = 2.1 Hz), 7.49 (m, 1H), 7.34 (m, 1H), 7.26 (s, 1H), 4.43 (t, 2H, J = 6.7 Hz), 1.83 (m, 2H), 1.53 (m, 2H), 1.02 (t, 3H, J = 7.4). Anal. calc. for $C_{15}H_{16}O_3$: C, 73.75%; H, 6.60%. Found: C, 73.64%; H, 6.73%. The spectral data agreed with previously reported data [1].

Cyclohexyl 3-hydroxy-2-naphthoate (30)

Colorless crystal. Yield: 34%. Mp: 220-222°C. ^{1}H NMR (300 MHz, $CDCl_3$, δ ppm): 1.4274-1.5294 (m, 6H), 1.7403-1.7632 (m, 2H), 1.9743-1.9922 (m, 2H), 4.6058-4.6289 (m, 1H), 7.3557 (dd, J1=1.52Hz, J2=7.66Hz, 1H), 7.6315 (dd, J1=1.65, J2=7.57, 1H). 7.8755-7.9034 (m, 3H), 8.2544 (s, 1H), 13.2686 (s, 1H). ESI MS: 271.13 $[M + Na]^+$. Anal. calc. for $C_{17}H_{18}O_3$: C, 75.53%; H, 6.71%, found: C, 75.35%; H, 6.78%.

Octyl 3-hydroxy-2-naphthoate (31)

Yellow crystal. Yield: 38%. Mp: 231-232°C. ^{1}H NMR (300 MHz, $CDCl_3$, δ ppm): 0.9421 (t, J=8.06Hz, 3H), 1.2559-1.4342 (m, 10H), 1.7645-1.7833 (m, 2H), 4.4395 (t, J=7.12Hz, 2H), 7.3639 (dd, J1=1.64Hz, J2=7.68Hz, 1H), 7.6437 (dd, J1=1.66Hz, J2=7.64Hz, 1H). 7.8622-7.8911 (m, 3H), 8.3414 (s, 1H), 14.2761 (s, 1H). ESI MS: 301.18 $[M + Na]^+$. Anal. calc. for $C_{19}H_{24}O_3$: C, 75.97%; H, 8.05%, found: C, 75.88%; H, 8.01%.

Table S1. Predicted and experimental pEC_{50} values of 3D-QSAR models

	M. aeruginosa		*M. wesenbergii*		*M. flos-aquae*		*Ap. flos-aquae*		*An. flos-aquae*	
Comp.	Exp. pEC_{50}	Pred. pEC_{50}	Exp. pEC_{50}	Pred. pEC_{50}	Exp. pEC_{50}	Pred. pEC_{50}	Exp. pEC_{50}	Pred. pEC_{50}	Exp. pEC_{50}	Pred. pEC_{50}
1	2.90577	2.93105	2.94455	3.09321	2.99965	2.90168	3	3.15095	3.56193	3.60292
2	3.15621	3.24808	3.15852	3.66031	3.35154	3.98284	3	3.48403	3.43616	3.76156
3	4.21098	4.26894	4.18709	4.11962	4.1343	4.13984	4.25956	4.01317	3.90868	3.8835
4	4.48812	4.56579	4.61798	4.66913	4.63451	4.6555	4.12407	4.40824	3.7948	4.15912
5	4.56225	4.5566	4.18776	4.09679	4.50169	4.53881	4.61798	4.27179	3	2.87556
6	4.56004	4.57809	4.96577	5.21466	5.13847	5.67398	4.26122	3.70537	3	2.7632
7	4.15255	3.98017	4.56479	4.14571	5.44491	4.56298	4.06905	4.25309	4.15745	3.7829
8	3	3.03601	3	3.32838	4.09691	3.93064	4.55284	4.70079	3	3.24071
9	3	3.19874	3	4.00383	3	3.30822	4.29619	4.92977	4.12131	4.1375
10	4.04915	3.90585	3.96658	4.03116	4.06048	4.15832	4.23062	4.04249	4.02743	3.98977
11	3	2.97592	4.20412	4.10703	3	3.26601	4.37572	4.51259	3	3.58874
12	3.93779	3.95096	3.96019	3.77079	3	2.81536	4.71897	4.53755	3.91578	3.80063
13	4.18349	4.10406	3.35205	3.57637	3.62342	3.78199	4.03255	4.33731	4.05154	3.65226
14	4.00174	3.97246	4.36231	3.96893	4.41341	3.92431	4.83893	4.73441	3.75259	3.62888
15	4.68719	4.5946	5.11182	5.05247	4.86328	5.02155	4.64359	4.45098	4.43309	3.9214
16	4.96981	4.7961	5.25493	5.07106	4.70752	4.56114	5.25259	5.34023	4.39903	4.58045
17	4.81645	4.72048	4.93219	4.83711	4.78331	4.77715	5.4045	5.06962	4.08847	3.92913
18	4.85605	4.81567	4.88041	4.59523	4.90101	4.63973	6.20066	5.90188	4.2136	4.30844
19	4.68173	4.63354	4.85981	5.23503	4.92885	4.83482	5.33819	5.608	4.51899	4.59128
20	4.7217	4.77747	4.72538	4.93166	4.84924	4.99222	4.82857	4.8828	4.08571	3.7976
21	4.61066	4.76694	4.85761	4.82229	4.92922	4.76351	5.21753	5.47051	4.24162	4.23281
22	4.09302	4.16246	3.83375	3.93541	4.14685	4.02378	4.04648	4.443	2.0167	2.42599
23	4.81559	5.01847	4.5558	4.23407	4.66434	4.55799	4.84285	4.53703	3.83726	3.99974
24	5.86012	5.85143	4.41319	4.28946	4.57545	4.55567	5.36754	5.19947	3.9426	3.37049
25	4.47057	4.32425	3.97634	4.23789	3	3.24275	4.20447	4.38347	2.78174	2.82868

26	4.49026	4.49852	4.33479	4.20288	3	3.08384	4.94348	4.82217	3.67178	3.61185
27	4.61137	4.74446	3.8707	3.66624	3	2.80033	5.0716	4.86049	3.64138	3.8141
28	4.21028	4.1764	3	2.96385	3	2.86233	4.08986	4.06508	3.92941	4.10918
29	4.54745	4.62134	4.35223	3.77858	3	2.87287	5.00305	4.53968	4.42033	4.10659
30	4.84345	5.02631	4.79915	4.64877	3	3.05967	5.59176	5.312	4.4176	4.36975
31	4.80967	4.69923	4.25885	3.88327	3	2.85971	5.22621	4.96444	3.64929	3.80177
CA	5.20901	4.92942	4.35655	4.24809	5.50446	5.28409	4.13077	3.9221	3.86012	3.81913
CIA	5.41229	5.55527	4.06611	4.3788	3.76976	3.97022	4.58336	4.89893	2.78174	2.97858
HNA	4.35813	4.29779	3.93115	4.04442	3	3.61872	4.17666	4.788	4.07242	4.26635

Supplementary references

1. Xia, C.-n.; Li, H.-b.; Hu, W.-x. Synthesis of trans-caffeate analogues and their bioactivities against HIV-1 integrase and cancer cell lines. *Bioorg Med Chem Lett* **2008**, *18*, 6553-6557.
2. Uwai, K.; Osanai, Y.; Imaizumi, T.; Kanno, S.; Takeshita, M.; Ishikawa, M. Inhibitory effect of the alkyl side chain of caffeic acid analogues on lipopolysaccharide-induced nitric oxide production in RAW264.7 macrophages. *Bioorg Med Chem* **2008**, *16*, 7795-7803, doi:10.1016/j.bmc.2008.07.006.
